# Prevalence of MRI lesions in men responding to a GP-led invitation for a prostate health check: a prospective cohort study

**DOI:** 10.1136/bmjonc-2023-000057

**Published:** 2023-08-21

**Authors:** Caroline M Moore, Elena Frangou, Neil McCartan, Aida Santaolalla, Douglas Kopcke, Giorgio Brembilla, Joanna Hadley, Francesco Giganti, Teresa Marsden, Mieke Van Hemelrijck, Fiona Gong, Alex Freeman, Aiman Haider, Steve Tuck, Nora Pashayan, Thomas Callender, Saran Green, Louise C Brown, Shonit Punwani, Mark Emberton

**Affiliations:** 1 Division of Surgery & Interventional Science, University College London, London, UK; 2 Urology, University College London Hospitals NHS Foundation Trust, London, UK; 3 MRC Clinical Trials Unit, University College London, London, UK; 4 Our Future Health, London, UK; 5 Translational Oncology and Urology Research (TOUR), Centre for Cancer, Society and Public Health, School of Cancer and Pharmaceutical Sciences, King's College London, London, UK; 6 Centre for Medical Imaging, University College London, London, UK; 7 Department of Radiology, University College London Hospitals NHS Foundation Trust, London, UK; 8 Department of Histopathology, University College London Hospitals NHS Foundation Trust, London, UK; 9 Oxfordshire Prostate Cancer Support Group, Oxford, UK; 10 Department of Applied Health Research, University College London, London, UK; 11 Division of Medicine, University College London, London, UK; 12 Faculty of Medical Sciences, University College London, London, UK

**Keywords:** Prostate cancer, Cancer screening

## Abstract

**Objective:**

In men with a raised prostate-specific antigen (PSA), MRI increases the detection of clinically significant cancer and reduces overdiagnosis, with fewer biopsies. MRI as a screening tool has not been assessed independently of PSA in a formal screening study. We report a systematic community-based assessment of the prevalence of prostate MRI lesions in an age-selected population.

**Methods and analysis:**

Men aged 50–75 were identified from participating general practice (GP) practices and randomly selected for invitation to a screening MRI and PSA. Men with a positive MRI or a raised PSA density (≥0.12 ng/mL^2^) were recommended for standard National Health Service (NHS) prostate cancer assessment.

**Results:**

Eight GP practices sent invitations to 2096 men. 457 men (22%) responded and 303 completed both screening tests. Older white men were most likely to respond to the invitation, with black men having 20% of the acceptance rate of white men.

One in six men (48/303 men, 16%) had a positive screening MRI, and an additional 1 in 20 men (16/303, 5%) had a raised PSA density alone. After NHS assessment, 29 men (9.6%) were diagnosed with clinically significant cancer and 3 men (1%) with clinically insignificant cancer.

Two in three men with a positive MRI, and more than half of men with clinically significant disease had a PSA <3 ng/mL.

**Conclusions:**

Prostate MRI may have value in screening independently of PSA. These data will allow modelling of the use of MRI as a primary screening tool to inform larger prostate cancer screening studies.

**Trial registration number:**

NCT04063566.

**WHAT IS ALREADY KNOWN ON THIS TOPIC**

- The European Randomised Screening for Prostate Cancer study used prostate-specific antigen (PSA) >3 ng/mL, or an abnormal digital rectal examination (DRE) to select men for a standard transrectal biopsy. The study reported a 20% reduction in prostate cancer mortality at 16 years but was associated with significant overdiagnosis and overtreatment.
- Replacing standard transrectal biopsy with prostate MRI, and targeted biopsy in men with an MRI lesion, in men who have a high PSA, or abnormal DRE allows at least 1 in 4 men to avoid unnecessary biopsy, and reduces overdiagnosis and overtreatment.

**WHAT THIS STUDY ADDS**

- We assess the prevalence of lesions on prostate MRI in men invited for a prostate health check. We found that 1 in 6 screened men had a lesion on MRI, and over half of the men with significant cancer on biopsy had a PSA <3 ng/mL. Less than 1% of screened men were ‘overdiagnosed’ with low-risk disease.

**HOW THIS STUDY MIGHT AFFECT RESEARCH PRACTICE OR POLICY**

- We should evaluate the use of an MRI-led approach to prostate cancer screening in a larger UK population, to assess whether it could maintain the reduction in prostate cancer mortality of formal screening, while reducing overdiagnosis and associated overtreatment by using an MRI-led approach.

## Introduction

Prostate cancer is the most common cancer, and the second most common cause of cancer-related death, in men in the UK.^1^ The UK, with no formal screening programme, has a high age-standardised prostate cancer-specific mortality, at 12.4/100 000 population, compared with the USA at 8.2, France 8.4, Spain 7.3 and Italy 5.9.^2^

The European Randomised Screening study for Prostate Cancer demonstrated that organised screening can reduce prostate cancer mortality, compared with controls, by 20% at 16-year follow-up.^3^ However, this comes at a significant cost of overdiagnosis. Screening using prostate-specific antigen (PSA) as triage and transrectal biopsy as verification resulted in half of all detected cancers being low grade and unlikely to result in a prostate cancer death but was still associated with acceptance of radical treatment. The Cluster Randomised Trial in over 400 000 men in the UK, using a single PSA test, reported similar prostate cancer-specific and all-cause mortality rates between screened men compared with controls when analysed at 10 years, but an increase in the proportion of men diagnosed with low-risk prostate cancer.^4^ Overdiagnosis, and the associated personal and economic costs of continued monitoring or ‘overtreatment’, has proved a significant barrier to the introduction of screening programmes based on PSA and standard transrectal biopsy.

A number of studies have shown that, in men with a raised PSA or abnormal digital rectal examination (DRE), an MRI scan between PSA and subsequent biopsy verification reduces unnecessary biopsy, and subsequent diagnosis of indolent disease; and by detecting more clinically significant disease than standard biopsy alone.^5–7^ Given the known inherent error associated with both PSA and traditional transrectal biopsy, the next question to ask was: ‘How would MRI perform on its own if used in an age defined—not PSA defined—population setting?’

In the PROMIS study, in a clinical population defined by raised PSA or abnormal DRE, it was found that over half of the significant cancers seen on MRI were missed on standard transrectal biopsy.^8^ MRI lesions scoring 4/5 had >50% likelihood of harbouring clinically significant cancer, and those scoring 5/5 had >70% likelihood of harbouring clinically significant cancer. MRI lesions are positively correlated with higher histological grade and prostate cancer volume.^9^ The study we report here allowed us to explore the prevalence of MRI lesions in men based on age, not PSA. This knowledge will permit us to both model the performance of an MRI-based screening strategy and design the next stage in exploring its role as a primary screening test.

### Objective

To report the prevalence of a positive screening MRI in men who respond to a general practice (GP)-led invitation for prostate cancer screening, to inform future prostate cancer screening strategies.

## Materials and methods

### Study design

The ReIMAGINE prostate cancer screening study was a prospective single-centre feasibility study designed to assess the feasibility of a screening approach using PSA and MRI.^10^ University College London (UCL) is the study sponsor (122665). This study is supported by the Medical Research Council (MRC) (grant number MR/R014043/1) and Cancer Research UK (CRUK). The study is registered at ClinicalTrials.gov.

### Setting

Participants were selected for invitation by participating GP practices, and the screening procedures (MRI and PSA) were carried out in a single London university hospital.

### Participants

Potential participants were identified through screening of existing patient databases at eight London GP surgeries. Men aged 50–75, without a prior prostate cancer diagnosis, were identified and randomly selected for invitation.

### Invitation

A letter explaining the study was sent, and men were invited to contact the study group to be assessed for eligibility. Eligible men who were keen to take part, having already received a patient information sheet, were offered a screening visit at University College London Hospital Trust.

### Screening assessment

The screening visit included the consent process. Consented men had a PSA blood test, and a screening MRI (sMRI). PSA density was calculated using sMRI-measured prostate volume.

The sMRI consisted of clinical and research-specific T2 exploratory acquisitions, carried out in a 3 Tesla scanner, with a total scan time of <20 min. The clinical sequences included T2-weighted axial turbo spin echo and diffusion-weighted imaging using a high *b* value of 2000 s/mm^2^ with an acquisition time under 10 min. Contrast enhancement was not used, and there were no apparent diffusion coefficient sequences.

The MRI was scored positive or negative by two radiologists independently, according to pre-defined criteria, with a third reviewer if the two radiologists were not in agreement on the screening result.

Men were deemed screen positive if they had a positive sMRI or a PSA density of >0.12 ng/mL^2^.^11^ Screen negative men were informed of their result, and exited the study. Screen positive men were told of their screening result, and recommended to have a referral for National Health Service (NHS) assessment on an urgent suspected cancer pathway. Biopsies were carried out if indicated after multiparametric MRI within a standard NHS pathway. The biopsies were done according to local practice at one of two London hospitals. A transperineal approach was used, with targeting of the MRI lesion and systematic sampling of the peripheral zones. They were followed up for the outcome of this assessment. They exited the study at this point, but gave permission for data to be collected from their standard of care NHS assessment. Clinically significant cancer was defined as any Gleason pattern 4 or above.

### Patient and public involvement (PPI)

Patients were involved in the planning and design of the research, and were interviewed with two senior researchers by the grant awarding committee, a key step in the process. Patients co-developed the screening protocol and suggested lowering the age of invitation to 50, due to concerns of missed significant cancers in younger men. When the study was paused for COVID-19 in April 2020, the PPI group were instrumental in designing modifications to allow continued recruitment with COVID-19-safety measures, leading to recruitment ahead of the original planned schedule.

### Statistical analysis

Data were accessed via the UCL Research Electronic Data Capture (REDCap) service. STATA V.16.1 was used throughout the analysis.

Age was reported within 5-year bands. Ethnicity was presented across broad categories. Summary statistics were used to describe data; mean (range, SD) or median (IQR) for continuous variables or n (%) for categorical variables. Proportions were reported alongside 95% CIs. Univariable and multivariable logistic regression was used to explore baseline characteristics in relation to acceptance rates and screening results. Results are presented as ORs with 95% CIs. ORs of higher than 1 indicate greater association between a baseline variable and the outcome; when the variable is categorical, the OR is interpreted with respect to the reference category which is stated in each case. Significance is assessed at the 5% level.

Partial postcodes and in particular postcode sectors were collected for all invited participants. GeoConvert^12^ was used to obtain full postcodes and subsequently Index of Multiple Deprivation (IMD) scores for each postcode. The IMD scores were then averaged and mapped back to the collected postcode sectors. Census data from 2011 were used.

### Role of the funding source

The MRC and CRUK had no role in the study design, data collection, data analysis, data interpretation or writing of the report. CM and EF had full access to the data and CM had final responsibility for the decision to submit for publication.

## Results

### Response to the screening invitation

**Figure 1:**
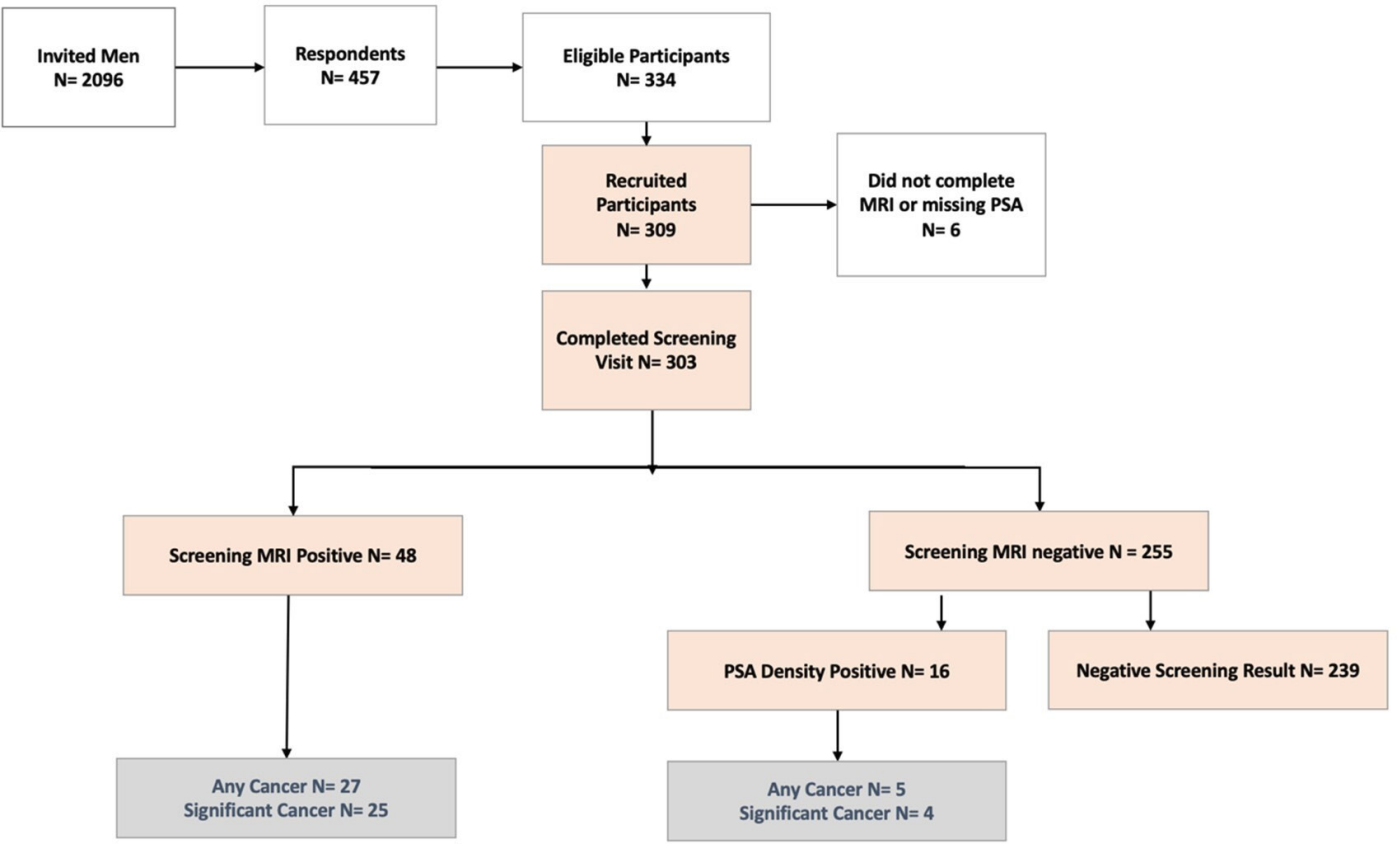
Study participant flowchart. PSA, prostate-specific antigen.

Two thousand and ninety-six men were invited across eight GP practices, and 457 men (22%) contacted the study team in response to the invitation. Of these, 309 men attended for screening (figure 1). The number of men screened was limited by the availability of a fixed number of MRI slots, so not all eligible responders were able to participate.

Baseline characteristics are shown in table 1. The mean age of recruited participants was 61.9 (range 50–77) and median PSA was 1.2 ng/mL (IQR 0.7–2.2).

**Table 1:** Baseline and screening visit characteristics

Variable at baseline	Total (n=309)
Years at screening	
Mean (SD)	61.9 (7.23)
Family history	
No	267 (86%)
Yes	41 (13%)
Missing	1 (0%)
Charlson Comorbidities Index	
1–2	229 (74%)
3–4	77 (25%)
+5	3 (1%)
PSA, Median (IQR), ng/mL	1.2 (0.7–2.2)
Prostate volume, Median (IQR), mL	29 (23–28)
PSA density, Median (IQR), ng/mL^2^	0.04 (0.03–0.06)
Screening MRI positive	48 (16%)
PSA density positive	16 (5%)
Screening visit negative	239 (77%)
Visit not completed	6 (2%)

PSA, prostate-specific antigen.

### Characteristics of responders versus non-responders

Logistic regression results show that age and ethnicity are both associated with response to screening invitation, with older white men the most likely to respond. Multivariable logistic regression showed that black men had one-fifth the response rate of white men. IMD did not vary between responders and non-responders (table 2). The ethnicity distribution of invited men reflected the ethnicity distribution across London (table 3).

**Table 2:** Logistic regression of screening invitation acceptance

Variable	Accepted invitation, n (%)* or Mean (SD)	OR (95% CI), p value	
	Yes	Univariable model	Multivariable model
Age bands			
50–55 (n=614 (34%))	115 (19)	0.69 (0.49 to 0.95), 0.023	0.71 (0.51 to 0.98), 0.039
55–60 (n=461 (25%))	87 (19)	0.69 (0.49 to 0.98), 0.037	0.70 (0.49 to 0.99), 0.044
60–65 (n=314 (17%))	79 (25)	Reference†	Reference†
65–70 (n=231 (13%))	74 (32)	1.40 (0.96 to 2.04), 0.078	1.36 (0.92 to 1.99), 0.120
>70 (n=180 (10%))	51 (28)	1.18 (0.78 to 1.78), 0.441	1.06 (0.70 to 1.61), 0.792
Missing (n=22 (1%))	10 (45)		
Ethnicity			
White (n=1099 (60%))	312 (28)	Reference‡	Reference‡
Black (n=141 (8%))	11 (8)	0.21 (0.11 to 0.40), <0.001	0.22 (0.12 to 0.42), <0.001
Asian (n=176 (10%))	20 (11)	0.32 (0.20 to 0.52), <0.001	0.33 (0.20 to 0.54), <0.001
Other (n=44 (2%))	8 (18)	0.56 (0.26 to 1.22), 0.144	0.55 (0.25 to 1.20), 0.134
Mixed (n=29 (2%))	7 (24)	0.80 (0.34 to 1.90), 0.616	0.86 (0.36 to 2.05), 0.735
Missing (n=332 (18%))	58 (17)	0.53 (0.39 to 0.73), <0.001	0.48 (0.34 to 0.67), <0.001
IMD score (n=1800 (99%)	406 (23), 19.8 (7.0)	0.99 (0.98 to 1.01), 0.335	1.00 (0.99 to 1.02), 0.817

*Total n is based on data from six GP practices (one practice did not provide data while another one only provided aggregate data) and is equal to 1821 invited men; missingness for the age band variable was n=21 (1%).

†The reference category for age is the 60-65 years band.

‡The reference category for ethnicity is ‘White’.

GP, general practice; IMD, Index of Multiple Deprivation.

**Table 3:** Ethnic distribution of London population versus ReIMAGINE

	London population, n=797 062	ReIMAGINE	
		Invited, n=1607*	Respondents, n=374†
Ethnicity			
White	569 308 (71%)	1140 (71%)	317 (85%)
Black	71 152 (9%)	196 (12%)	15 (4%)
Asian	112 260 (14%)	178 (11%)	20 (5%)
Mixed	13 213 (2%)	39 (2%)	8 (2%)
Other	13 572 (2%)	54 (3%)	14 (4%)

White: British, Irish, other white.

Asian: Indian, Pakistani, Bangladeshi, other Asian.

Other: other.

Mixed: Caribbean, African, Asian, other mixed.

*Missing ethnicity data 490/2097 (23%) in the ReIMAGINE invited individuals.

†Missing ethnicity data 83/457 (18%) in the ReIMAGINE respondents.

### Results of screening tests

#### Prevalence of a positive MRI

**Figure 2:**
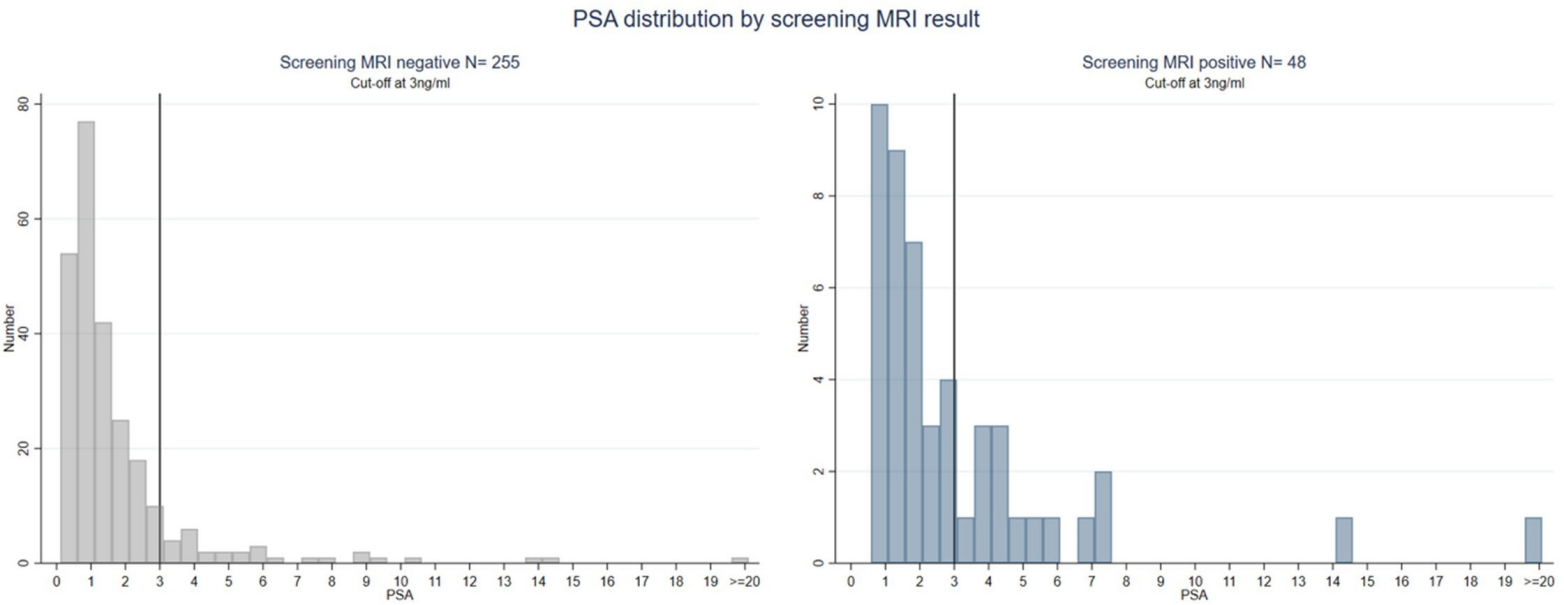
Histogram of PSA distribution according to MRI result. PSA, prostate-specific antigen.

Of 303 men who had an sMRI, 48 men (16%) had a lesion which was deemed screen positive (figure 1). Their median PSA was 1.2 ng/mL (IQR 0.7–2.2). Thirty-two of these 48 men (67%) had a PSA below 3 ng/mL (figure 2). None of the 13 black men in the study had a positive MRI (online supplemental table A1). Logistic regression by age, ethnicity and IMD showed that older age and Asian or other ethnicity were associated with a higher likelihood of a positive MRI (online supplemental table A2).

10.1136/bmjonc-2023-000057.supp1 Supplementary data

#### PSA density results

Of the 255 men with a negative MRI, 16 men (5%) had a PSA density of >0.12 ng/mL^2^, and were also recommended to have an NHS referral for further assessment. Three of these 16 men (19%) had a PSA below 3 ng/mL. Logistic regression showed that PSA density was significantly higher in black and Asian men, and in men aged 65–70 (online supplemental table A3).

#### Referral for NHS assessment

Sixty-four of 303 men (21%) had either a positive screening MRI or a raised PSA density and were recommended for further assessment via the NHS. These referrals and assessments were done according to local GP practice preference and were outside of the study protocol. Men consented for these data to be collected.

#### Outcome of NHS assessment

Of the 48 men with a positive screening MRI, 25 (52%) had clinically significant cancer, and 2 men (4%) had clinically insignificant cancer. The full biopsy characteristics, MRI lesion volume and PSA of all men with detected cancers are shown in online supplemental table A4.

The biopsy characteristics of the cancers detected in 17 men with a positive screening MRI and a PSA <3 ng/mL included 2 Gleason 3+3 cancers, 13 Gleason 3+4 cancers, with a mean cancer core length (MCCL) of 7 mm, 1 Gleason 4+3 (3mm MCCL) and 1 Gleason 4+5 (9mm MCCL) (online supplemental table A4).

Of the 25 men with a positive MRI and clinically significant cancer, 15 had a PSA <3 ng/mL.

Of the additional 16 men who had a negative MRI but a raised PSA density, 4 men (25%) had clinically significant cancer and 1 man (6%) had a clinically insignificant cancer.

## Discussion

### Summary of results

In response to a single paper invitation for screening, 457 of 2096 men (22%) responded. Older men were more likely to respond to the invitation, and multivariable logistic regression showed that black men had one-fifth the response to invitation compared with white men. Not all men who responded to the invitation were able to take part, as funding limited the number of available MRI slots, and men were allocated on a ‘first come, first served’ basis.

Of 303 men who had both screening tests, 64 (21%) screened positive and were recommended for referral for further NHS assessment, outside of the study. One in 6 men (48 of 303, 16%) had a screen positive MRI, and an additional 1 in 20 men (16 of 303, 5%) tested positive on PSA density alone. Two-thirds (32/48) of men with a screen positive MRI had a PSA below 3 ng/mL, and over half of men (15/25) with a positive MRI and clinically significant cancer had a PSA below 3 ng/mL.

After NHS assessment outside of the study, 29 of 303 screened men (9.6%) had clinically significant disease and 3 of 303 men (1%) had clinically insignificant disease.

### Limitations of the study

This feasibility study was carried out in a small sample of men across a number of different London GP practices, nominated as research active practices by Noclor, a research support service for primary care. As the scanning centre was based in London, it was not practical for invitations to be sent more widely. A single paper invitation was sent. Formal UK screening programmes would also include more general measures such as advertising campaigns which are likely to increase recruitment. The ethnicity distribution of invited men was reflective of the ethnicity distribution of London as a whole (table 3).

The study started prior to the COVID-19 pandemic, paused recruitment from April to August 2020, and then restarted. At the time, many people were avoiding visits to healthcare facilities, and our PPI panel developed a number of strategies to address the COVID-19 concerns of potential participants.^13^ These strategies included a dedicated cleaning schedule between patients, ensuring that the participant did not come into contact with other participants, or hospital patients during their visit, and that private transport by car was funded for participants. Even so, the response to the invitation is likely to have been impacted by the pandemic.

Study participation was completed when the results of the screening tests were given, with further assessment, including biopsy if needed, done via the usual NHS pathway. This follows the pattern of formal screening programmes in breast, colorectal and cervical cancer in the UK, although differs from other prostate cancer screening studies which included biopsy within the study protocol.

### Clinical implications

MRI as a triage test between a raised PSA or an abnormal DRE, and a biopsy, has been recommended in UK guidelines since 2018.^14 15^ It is now also recommended in the European Association of Urology guidelines,^16^ and by the American Urology Association^17^

In clinical practice, we recognise that the PSA test has limitations in the identification of men at risk for prostate cancer. MRI may allow us an alternative way to assess prostate cancer risk in men in the community. Normative data on the prevalence of MRI lesions in an age-defined systematically recruited community-based population has not been previously reported. The PROSTAGRAM study used MRI, ultrasound and PSA to assess men, but had a mixed approach to recruitment using both invitation via GP practices and some approaches directly to the black community in London.^18^ Nam and colleagues in Toronto recruited men to an MRI-based screening assessment using a newspaper advert, and responders may not be representative of the population who would be invited for screening using formal healthcare-based mechanisms.^19^

The finding in this report is that 2 in 3 men with a positive screening MRI have a PSA <3 ng/mL is a sobering one because MRI lesions are positively associated with clinically significant cancer.

Using MRI to detect cancers can allow pick up of significant lesions before the PSA has begun to rise, and so offer an opportunity for early detection. It could lead to ‘overdetection’ of cancers that will not become clinically relevant in a patient’s lifetime. Subsequent screening of the same men after an appropriate time interval would enable us to see whether this high prevalence on first screen is balanced by lower detection on re-screening.

In traditional PSA triage-based screening, these men would have tested negative and have been reassured. This observation might explain why a single PSA-based screening confers so little impact on prostate cancer-specific and all-cause mortality. The more recent work of Eklund and colleagues used a PSA cut-off of 1.6 ng/mL for further assessment, and this may be a more appropriate approach,^7^ although the use of PSA and MRI as independent risk assessment tools should be explored further in a larger screening study.

### Future research

This study has been carried out in a hospital-based setting, at a single university hospital. A screening programme would need to be delivered at specialised screening centres, where consistent high quality acquisition and reporting would need to be achieved.^20^ Future research would need to assess the feasibility of a community-based MRI delivery programme, with use of a mobile MRI scanner, such as those used in some breast screening programmes.

A response rate of 22% for a single paper-based invitation, during a global pandemic when people were discouraged from attending healthcare settings, is likely to increase in non-pandemic times. The differential response rate, based on age and ethnicity, needs to be addressed. In terms of age, those most likely to respond were those in the 65–70 age band, which has a higher prostate cancer incidence than younger men. In terms of ethnicity, black men were the least likely to respond to an invitation, but have a higher risk of prostate cancer than white men.

In an ideal situation, the likelihood of response to a screening invitation would be proportionate to the risk of disease in that group. A recent model-based analysis, based on USA SEER data, suggested that increasing the intensity of PSA screening in black men between the ages of 45 and 70 would lead to a greater mortality reduction, and limited overdiagnosis, compared with historical general population screening.^21^ In order to design a screening study which targets men at highest risk of prostate cancer, a variety of approaches may be needed. A recent initiative by Orchid, a UK-based men’s health charity explored ways to engage black and Afro-Caribbean men either diagnosed or at risk of prostate cancer.^22^ Successful approaches including raising awareness among men and women in the community through roadshows and dedicated materials including a short film, and z-cards with relevant information. It also included increasing awareness among healthcare professionals about the increased risk of prostate cancer in black men.

Further screening studies would need to incorporate other prostate cancer risk assessment approaches including non-imaging biomarkers, to assess the most efficient screening approach in the UK population. Given the incomplete overlap of the risk profiles generated by PSA and MRI, we would encourage each to be used in further research, to assess whether a stepwise approach can be adopted.

## Data Availability

Data are available upon reasonable request. The datasets generated for this study can be found in the ReIMAGINE instance of the Philips HSDP CDL at https://research-cdl-prod-cdlux.eu-west.philips-healthsuite.com/catalog. These datasets are currently accessible only to the consortium partners of the ReIMAGINE project. After completion of the project, the data will also be available to the broader clinical and scientific community via request to the ReIMAGINE Group (reimagine@ucl.ac.uk). All data released (Individual participant data (including data dictionaries)) from the consortium will appear in an anonymised format. The study protocol, statistical analysis plan, and informed consent will be available after publication.
