# Supplementary material for: Prevalence of MRI lesions in men responding to a GP-led invitation for a prostate health check: a prospective cohort study
Source: BMJ Oncol. 2023 Aug 21;2(1):e000057. doi: 10.1136/bmjonc-2023-000057 (PMC11315271; doi:10.1136/bmjonc-2023-000057)
Supplement: Supplementary data [file bmjonc-2023-000057supp001.pdf]

Appendix Material

Table A1 Screening visit result by ethnicity category

Table A2 Logistic regression of MRI positivity by age, ethnicity and IMD

Table A3 Logistic regression of PSA density by age, ethnicity and IMD

Table A4 Biopsy characteristics of cancers picked up after NHS evaluation

Table A1: Screening visit result by ethnicity category

Broad Ethnicity Category	Screening MRI Positive N= 48	PSA Density Positive N= 16	Negative screening result N= 239	TOTAL N=303
**White**	43 (90%)	12 (75%)	204 (85%)	259 (85%)
**Black**	0 (0%)	3 (19%)	10 (4%)	13 (5%)
**Asian**	2 (4%)	1 (6%)	7 (3%)	10 (3%)
**Other**	3 (6%)	0 (0%)	12 (5%)	15 (5%)
**Not stated**	0 (0%)	0 (0%)	6 (3%)	6 (2%)

Table A2: Logistic regression of positivity on MRI N= 48

	Screening MRI status N, Mean (SD) or %	OR (95% C.I.), p-value	
**Variable**	**Positive**	**Univariable Model**	**Multivariable Model**
**Age bands**			
50-55 (N= 62 (20%))	2 (3%)	0.11 (0.02, 0.53), 0.005	0.12 (0.025, 0.55), 0.007
55-60 (N= 52 (17%))	3 (6%)	0.21 (0.06, 0.78), 0.019	0.20 (0.05, 0.76), 0.018
60-65 (N= 57 (19%))	13 (23%)	Reference	Reference
65-70 (48 (16%))	10 (21%)	0.89 (0.35, 2.26), 0.808	0.86 (0.33, 2.26), 0.763
>70 (N= 50 (17%))	13 (26%)	1.19 (0.49, 2.88), 0.701	1.08 (0.43, 2.70), 0.866
Missing (N= 34 (11%))	7 (21%)	-	-
**Ethnicity**			
White (N= 259 (86%))	43 (17%)	Reference	Reference
Black (N= 13 (4%))	-	-	-
Asian (N= 10 (3%))	2 (20%)	1.26 (0.26, 6.12), 0.778	2.26 (0.38, 13.41), 0.370
Other (N= 15 (5%))	3 (20%)	1.26 (0.34, 4.64), 0.733	1.07 (0.21, 5.56), 0.933
Not stated (N= 6 (2%))	-	-	-
**IMD score (N= 301 (99%))**	48 (16%), 18.7 (10.7)	0.99 (0.96, 1.02), 0.378	0.99 (0.96, 1.02), 0.518
**Family history**			
No (N= 262 (87%))	41 (16%)	Reference	Reference
Yes (N= 40 (13%))	7 (18%)	1.14 (0.47, 2.76), 0.766	1.29 (0.47, 3.58), 0.624
Missing (N= 1 (<1%))	-	-	-

Table A3: Logistic regression of positivity on PSA density alone N= 16

Variable	PSA density positive N, Mean (SD) or %	OR (95% C.I.), p-value Univariable Model	Multivariable Model
**Age bands**			
50-55 (N= 62 (20%))	2 (3%)	0.60 (0.10, 2.72), 0.584	0.80 (0.12, 5.37), 0.822
55-60 (N= 52 (17%))	3 (6%)	1.10 (0.21, 5.72), 0.908	1.34 (0.23, 7.69), 0.745
60-65 (N= 57 (19%))	4 (7%)	Reference	Reference
65-70 (48 (16%))	4 (8%)	1.64 (0.35, 7.71), 0.533	2.02 (0.40, 10.33), 0.398
>70 (N= 50 (17%))	1 (2%)	0.37 (0.04, 3.65), 0.393	0.54 (0.05, 5.85), 0.609
Missing (N= 34 (11%))	3 (9%)	-	-
**Ethnicity**			
White (N= 259 (86%))	12 (5%)	Reference	Reference
Black (N= 13 (4%))	3 (2%)	6.18 (1.50, 25.40), 0.012	6.95 (1.39, 34.68), 0.018
Asian (N= 10 (3%))	1 (10%)	2.29 (0.27, 19.55) 0.450	-[1]
Other (N= 15 (5%))	-	-	-
Not stated (N= 6 (2%))	-	-	-
**IMD score (N= 301 (99%))**	16 (5%), 18.6 (12.2)	0.99 (0.94, 1.03), 0.605	1.00 (0.95, 1.05), 0.893
**Family history**			
0 (N= 262 (87%))	16 (6%)	Reference	Reference
1 (N= 40 (13%))	-	-	-
Missing (N= 1 (<1%))	-	-	-

1: There is no OR for the Asian male as his age was missing so he was excluded from the multivariable model

Table A4 Biopsy characteristics of cancers picked up after NHS evaluation

i. Characteristics of cancer cases (non-significant cancer highlighted in grey) which had a positive MRI at the screening visit – Any cancer: N= 27/48 (56%), Significant Cancer N= 25/48 (52%)						
**Index**	**Overall Gleason score**	**Maximal Gleason score**	**% pattern 4**	**Maximum cancer core length (MCCL) across areas biopsed**	**PSA**	**PSA density**
1	G3+3	G3+3		.5	1.16	0.05
2	G3+3	G3+3		2	2.73	0.09
3	G3+4	G3+4	10	12	.85	0.02
4	G3+4	G3+4	10	3	.9	0.03
5	G3+4	G3+4	5	8	1.03	0.04
6	G3+4	G3+4	10	8	1.06	0.05
7	G3+4	G3+4	10	5	1.29	0.03
8	G3+4	G3+4	10	3	1.71	0.04
9	G3+4	G3+4	20	10	1.84	0.05
10	G3+4	G3+4	20	7	1.89	0.06
11	G3+4	G3+4	20	8	1.97	0.06
12	G3+4	G3+4	10	6	2.4	0.10
13	G3+4	G3+4	10	6	2.42	0.10
14	G3+4	G3+4	10	6	2.75	0.21
15	G3+4	G3+4	20	10	2.98	0.10
16	G3+4	G3+4	10	4	3.34	0.09
17	G3+4	G3+4	10	2	3.61	0.10
18	G3+4	G3+4	10	5	3.72	0.08
19	G3+4	G3+4	30	6	4.11	0.13
20	G3+4	G3+4	Not reported	9	5.33	0.15
21	G3+4	G3+4	10	9	5.68	0.16
22	G4+3	G4+3		3	1.	0.07
23	G4+3	G4+3		Not reported	4.12	0.21
24	G4+5	G4+5		9	2.04	0.23
25	G4+5	G4+5		12	4.97	0.18
26	G4+5	G4+5		12	7.53	0.33
27	G4+5	G4+5		8	43.5	0.75

ii. Characteristics of cancer cases (non-significant cancer highlighted in grey) deemed as screen positive on PSA density – Any cancer: N= 5/16 (31%), Significant Cancer N= 4/16 (25%)						
**Index**	**Overall Gleason score**	**Maximal Gleason score**	**% pattern 4**	**Maximum cancer core length (MCCL) across areas biopsed**	**PSA**	**PSA density**
1	G3+3	G3+3		1	2.98	0.12
2	G3+4	G3+4	20	1	3.79	0.13
3	G3+4	G3+4	40	7	9.22	0.26
4	G3+4	G3+4	10	12	10.5	0.23
5	G4+3	G4+3		6	6.42	0.27

iii. Volume of MRI lesions for those cases who were referred for NHS assessment (non-significant cancer highlighted in grey, no cancer in blue and no biopsy performed in yellow) deemed as screen positive on MRI – Any cancer: N= 31/48 (65%), Significant Cancer N= 29/48 (60%)				
**Index**	**Overall Gleason score**	**Volume of lesion 1**	**Volume of lesion 2**	**Volume of lesion 3**
1	G3+3	0.20	0.10	0.40
2	G3+3	2.00	.	.
3	G3+3	0.25	.	.
4	G3+4	0.25	.	.
5	G3+4	0.50	.	.
6	G3+4	0.30	.	.
7	G3+4	0.50	0.05	.
8	G3+4	0.15	.	.
9	G3+4	0.60	0.10	.
10	G3+4	0.30	.	.
11	G3+4	0.07	0.10	.
12	G3+4	0.10	0.05	.
13	G3+4	0.40	0.15	.
14	G3+4	2.50	.	.
15	G3+4	0.38	0.07	.
16	G3+4	0.25	.	.
17	G3+4	3.00	.	.
18	G3+4	0.06	0.07	.
19	G3+4	0.20	0.20	.
20	G3+4	0.09	0.70	.
21	G3+4	0.10	.	.
22	G3+4	0.35	.	.
23	G3+4	0.70	.	.
24	G3+4	0.15	.	.
25	G3+4	0.30	.	.
26	G4+3	0.30	0.08	.
27	G4+3	0.10	0.20	.
28	G4+5	3.50	.	.
29	G4+5	0.20	.	.
30	G4+5	15.00	.	.
31	G4+5	1.00	0.10	.
32	No cancer	0.50	.	.
33	No cancer	0.20	.	.
34	No cancer	0.90	.	.
35	No cancer	0.10	.	.
36	No biopsy performed	0.20	.	.
37	No biopsy performed	0.60	.	.
38	No biopsy performed	0.30	.	.
39	No biopsy performed	0.38	.	.
40	No biopsy performed	0.80	.	.
41	No biopsy performed	0.30	.	.
42	No biopsy performed	0.15	0.15	.

iii. Volume of MRI lesions for those cases who were referred for NHS assessment (non-significant cancer highlighted in grey, no cancer in blue and no biopsy performed in yellow) deemed as screen positive on MRI – Any cancer: N= 31/48 (65%), Significant Cancer N= 29/48 (60%)				
**Index**	**Overall Gleason score**	**Volume of lesion 1**	**Volume of lesion 2**	**Volume of lesion 3**
43	No biopsy performed	0.20	.	.
44	No biopsy performed	0.52	0.03	.
45	No biopsy performed	0.50	.	.
46	No biopsy performed	0.10	.	.
47	No biopsy performed	0.15	.	.
48	No biopsy performed	0.20	.	.

iv. MCCL of each area biopsied (non-significant cancer highlighted in grey, no cancer in blue) N= 37/64 (58%)						
**Index**	**Overall Gleason score**	**MCCL of biopsy area 1**	**MCCL of biopsy area 2**	**MCCL of biopsy area 3**	**MCCL of biopsy area 4**	**Maximum MCCL**
1	G3+3	.	0.50	.	.	0.50
2	G3+3	1.00	.	.	.	1.00
3	G3+3	.	1.00	2.00	.	2.00
4	G3+4	7.00	.	.	.	7.00
5	G3+4	6.00	0.50	.	.	6.00
6	G3+4	5.00	.	.	3.00	5.00
7	G3+4	9.00	1.50	8.00	.	9.00
8	G3+4	7.00	.	.	.	7.00
9	G3+4	1.00	2.00	.	.	2.00
10	G3+4	6.00	4.00	.	.	6.00
11	G3+4	.	1.00	.	.	1.00
12	G3+4	10.00	.	1.00	.	10.00
13	G3+4	6.00	2.00	.	.	6.00
14	G3+4	3.00	.	.	.	3.00
15	G3+4	6.00	.	.	.	6.00
16	G3+4	8.00	.	.	.	8.00
17	G3+4	5.00	5.00	.	.	5.00
18	G3+4	12.00	.	.	.	12.00
19	G3+4	12.00	.	.	.	12.00
20	G3+4	9.00	.	.	.	9.00
21	G3+4	4.00	.	.	.	4.00
22	G3+4	6.00	.	.	.	6.00
23	G3+4	10.00	.	.	.	10.00
24	G3+4	3.00	.	.	.	3.00
25	G3+4	8.00	5.00	.	.	8.00
26	G3+4	8.00	.	.	.	8.00
27	G4+3	.	.	.	.	.
28	G4+3	.	3.00	.	.	3.00
29	G4+5	12.00	1.00	.	.	12.00
30	G4+5	8.00	.	1.00	.	8.00
31	G4+5	12.00	.	.	.	12.00

iv. MCCL of each area biopsied (non-significant cancer highlighted in grey, no cancer in blue) N= 37/64 (58%)						
**Index**	**Overall Gleason score**	**MCCL of biopsy area 1**	**MCCL of biopsy area 2**	**MCCL of biopsy area 3**	**MCCL of biopsy area 4**	**Maximum MCCL**
32	G4+5	9.00	.	.	.	9.00
33	No cancer	.	.	.	.	.
34	No cancer	.	.	.	.	.
35	No cancer	.	.	.	.	.
36	No cancer	.	.	.	.	.
37	No cancer	.	.	.	.	.
